# Virtual first: implementation of a novel sleep telehealth platform in the United States military

**DOI:** 10.3389/frsle.2024.1304743

**Published:** 2024-02-08

**Authors:** Emerson M. Wickwire, Jacob Collen, Vincent F. Capaldi, Samson Z. Assefa, Rachell Jones, Scott G. Williams, Connie L. Thomas, Daniel C. Williams, Jennifer S. Albrecht

**Affiliations:** ^1^Sleep Disorders Center, Division of Pulmonary and Critical Care Medicine, Department of Medicine, University of Maryland School of Medicine, Baltimore, MD, United States; ^2^Department of Psychiatry, University of Maryland School of Medicine, Baltimore, MD, United States; ^3^Sleep Disorders Center, Walter Reed National Military Medical Center, Bethesda, MD, United States; ^4^Department of Medicine, Uniformed Services University of the Health Sciences, Bethesda, MD, United States; ^5^Department of Psychiatry, Uniformed Services University of the Health Sciences, Bethesda, MD, United States; ^6^Sleep Disorders Center, Alexander T. Augusta Military Medical Center, Fort Belvoir, VA, United States; ^7^Behavioral Biology Branch, Center for Military Psychiatry and Neuroscience, Walter Reed Army Institute of Research, Silver Spring, MD, United States; ^8^Center for Military Psychiatry and Neuroscience, Walter Reed Army Institute of Research, Silver Spring, MD, United States; ^9^Department of Family and Community Medicine, University of New Mexico School of Medicine, Albuquerque, NM, United States; ^10^Department of Epidemiology and Public Health, University of Maryland School of Medicine, Baltimore, MD, United States

**Keywords:** sleep, telehealth, implementation, insomnia, sleep apnea, military

## Abstract

**Background:**

There is a gross shortage of sleep specialist providers within the military health system. Telehealth and mobile health represent promising approaches to increase access to high quality, cost-effective care in the U.S military.

**Objectives:**

This paper reports findings from a mixed-methods clinical implementation study of a novel sleep telehealth platform at two military treatment facilities in the National Capitol Region. The platform includes a mobile app and integrated wearable sensors (i.e., a commercial off-the-shelf sleep tracker [Fitbit]). The primary purpose was to evaluate the implementation of a 10-day remote monitoring assessment and provision of evidence-based sleep treatment recommendations to patients and providers. In addition, we sought to observe, in an exploratory manner, subsequent engagement with the app during 5 days of personalized sleep education and training.

**Methods:**

Patients with sleep problems completed an intensive 10-day remote monitoring assessment that included a baseline intake questionnaire, daily sleep diaries, twice daily symptom surveys, and Fitbit. Based on this assessment, patients received personalized assessment results. Concurrently, a provider report was generated that included provisional diagnoses and evidence-based treatment recommendations. Next, participants gained access to personalized sleep education and trainings within the mobile app. Within an established implementation science framework, outcomes were assessed via behavioral adherence (engagement with the app) and separate questionnaires for patients and providers. Last, we conducted four focus groups with patients and 12 key informant interviews with primary care managers (PCMs) and economic stakeholders to seek feedback and recommendations for future directions.

**Results:**

Two hundred and seventy patients participated in the study. Using validated research questionnaires, participants reported high-risk for obstructive sleep apnea (65.6%), moderate to severe insomnia (38.2%), and moderate to severe daytime sleepiness (38.5%), and moderate to severe anxiety (14.1%) and depressive (20.4%) symptoms. Total sleep time was 6.6 (SD = 1.8) h based on sleep diaries and 6.1 (SD = 1.8) h based on Fitbit. Regarding implementation, reach, effectiveness, adoption, implementation, and maintenance were all notably high, based on quantitative and qualitative data from participants and PCMs.

**Conclusions:**

Sleep telehealth and mobile health represent promising approaches to increase access to cost-effective, evidence-based care for sleep disorders in the U.S. military.

## Introduction

Sleep is increasingly recognized as an important determinant of human health and performance. Due to an unrelenting tempo, non-traditional work hours, and deployments, insufficient and disturbed sleep are common in the U.S. military. Relative to civilians, active duty-service members (ADSMs) are far less likely to obtain seven or more hours of sleep per night, the minimum amount recommended for optimal health and daytime function among adults (28–32% vs. >70%) (Krueger and Friedman, 2009; Luxton et al., 2011; Mysliwiec et al., 2013; Hirshkowitz et al., 2015; Watson et al., 2015). Beyond insufficient sleep, clinical sleep disorders such as obstructive sleep apnea (OSA), insomnia, shift work, and nightmares are common and increasing (Williams et al., 2014; Capaldi et al., 2019; Devine et al., 2020). Between 2005 and 2019, the prevalence of OSA and insomnia increased from 11 to 333 per 10,000 ADSMs and from 6 to 272 per 10,000 ADSMs, respectively, perhaps in part due to increased awareness among providers of the health and performance consequences of sleep disorders (Moore et al., 2021). Left untreated, these conditions are associated with numerous adverse physical and mental health outcomes and reduced quality of life, resulting in diminished military readiness and dramatically increased economic costs (Williams et al., 2014; Wickwire et al., 2016, 2019; Capaldi et al., 2019; Devine et al., 2020).

There are well-recognized barriers limiting access to high-quality sleep medicine care within the U.S. military health system (MHS). Chief among these barriers is an insufficient number of trained specialist providers and relative dearth of accredited sleep centers. Currently, there are fewer than 30 board-certified sleep medicine physicians (18 Army, eight Air Force, four Navy) and fewer than 20 sleep centers (12 Army, three Air Force, two Navy) throughout all branches of the U.S. Armed Forces. Given the rapidly increasing awareness of sleep disorders in the MHS, clinical demand has rapidly outpaced the ability of the MHS to deliver high-quality sleep medicine care. Further straining these limited resources, traditional approaches to sleep care delivery can be time and resource intensive. For example, evaluation and management of most common sleep disorders (e.g., OSA and insomnia) typically requires multiple face-to-face treatment sessions that can be difficult to fit into military work schedules. Continuity of care is also challenging due to multiple moves every 1–3 years, temporary duty assignments, and deployments.

In the broader healthcare landscape, telehealth approaches including telemedicine (i.e., remote consultation via secure videoconference), internet/mobile health (i.e., web-based and app-based care), and wearables all represent promising potential solutions to help increase access to high-quality care. In sleep medicine in particular, telehealth approaches have demonstrated clinical non-inferiority and enhanced cost-effectiveness relative to traditional care. Yet telehealth remains underutilized in the MHS, a trend the Defense Health Agency (DHA) seeks to reverse. Indeed, under the direction of Army Lieutenant General Dr. Telita Crosland, the Defense Health Agency (DHA) has launched a major “virtual first”/“virtual front door” telehealth initiative to make military medicine “more precise, personal, predictive, preventive, and participatory” (News HI)^1^. Consistent with this approach, our group recently engaged, for the first time, key military stakeholders including patients, primary care manages (PCMs), and economic decision-makers in qualitative focus groups and key informant interviews designed to identify barriers and facilitators to sleep telehealth in the MHS. These stakeholders identified opportunities and provided recommendations to implement sleep telehealth via a novel platform designed to improve outcomes for all stakeholders (Abdelwadoud et al., 2021; Wickwire et al., 2022). This paper presents results of implementation of this novel sleep telehealth platform in two military treatment facilities (MTFs) in the National Capital Region (NCR).

## Materials and methods

### Study design and overview

This study employed a sequential mixed-methods (qualitative [QUAL 1]—quantitative [QUANT]—qualitative [QUAL 2]) design driven by continuous engagement with stakeholders including patients, primary care managers (PCMs), and economic stakeholders. The initial qualitative phase included focus groups with patients with sleep problems as well as one-on-one key informant interviews with PCMs and economic stakeholders; research methods and results have been presented elsewhere (Abdelwadoud et al., 2021; Wickwire et al., 2022). This paper presents methods and results from the quantitative and final qualitative phases.

In the quantitative phase (study 1, below), the novel sleep telehealth platform was implemented at two MTFs in the NCR. The primary purpose of this study was to evaluate the implementation of a ten-day remote monitoring assessment and provision of evidence-based sleep treatment recommendations to patients and providers. In addition, we sought to observe, in an exploratory manner, subsequent engagement with the app during 5 days of personalized sleep education and training. During the ten-day intensive remote monitoring period, participants completed a sleep history intake questionnaire via secure mobile app, wore a commercial off-the-shelf sleep tracker, completed daily sleep diaries, and completed brief symptom surveys via smart phone twice daily (i.e., 20 survey administrations across 10 days). Participants then received a report of assessment results that included evidence-based treatment recommendations, as well as personalized sleep education and daily training based on assessment results. In parallel, an assessment report was generated for providers that included provisional diagnoses and recommended next steps including evidence-based treatment recommendations. Following the 10-day remote monitoring period, patients received additional personalized sleep education materials within the app each day for 5 days and then completed an outcomes assessment. The final qualitative phase (study 2, below) included four focus groups with patients with sleep problems as well as 12 one-on-one key informant interviews with PCMs and economic stakeholders. Throughout the entire study, all participants received ongoing, routine clinical care within the MHS (e.g., including sleep diagnostic testing and treatment as determined by their health provider). This study was approved by the Institutional Review Board at Walter Reed National Military Center ([WRNMMC]; WRNMMC-2019-0258).

## Study 1

### Participants

#### Patients with sleep problems

Participants were recruited from the Internal Medicine clinic and the Sleep Disorders Center at WRNMMC and Family Medicine clinic and Sleep Disorders Center at the Alexander T. Augusta Military Medical Center (ATAMMC, formerly Ft. Belvoir Community Hospital). Active duty military servicemembers and/or Defense Eligibility Enrollment System beneficiaries between the ages of 18–75 years were eligible to participate. Additional inclusion criteria included ownership of a smartphone and provider or self-referral for sleep problems (including insufficient sleep). Exclusion criteria included pregnancy, untreated and/or uncontrolled medical or psychiatric illness, and pending retirement or permanent international change of station.

#### Healthcare providers

Healthcare providers (primary care managers [PCMs]) were recruited via word of mouth from the Internal Medicine clinic at WRNMMC and Family Medicine clinic at ATAMMC.

#### Sleep telehealth platform

**Figure 1:**
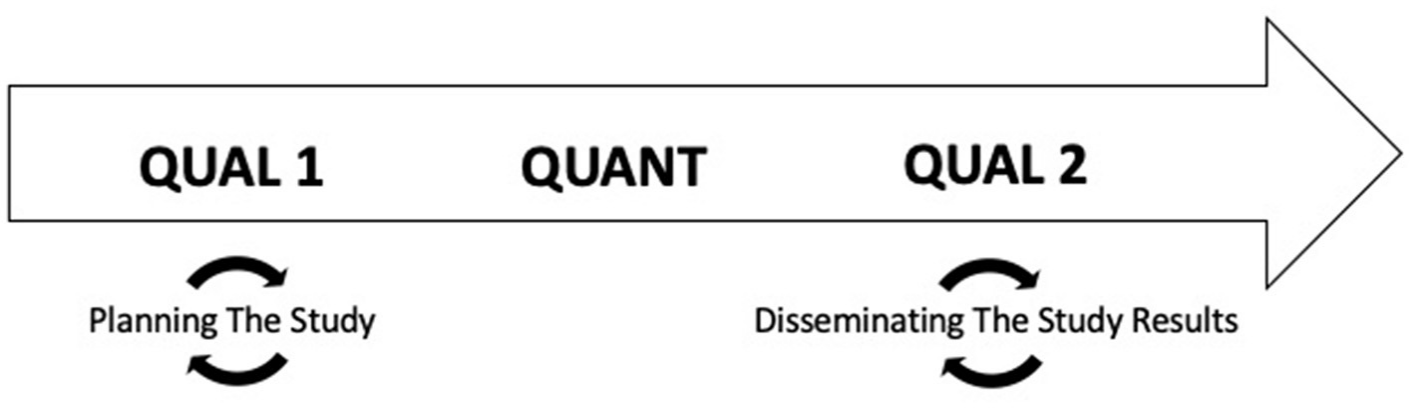
Study design. This study employed a sequential exploratory mixed-methods study design driven by continuous engagement with stakeholders including patients, primary care managers (PCMs), and economic stakeholders.

**Figure 2:**
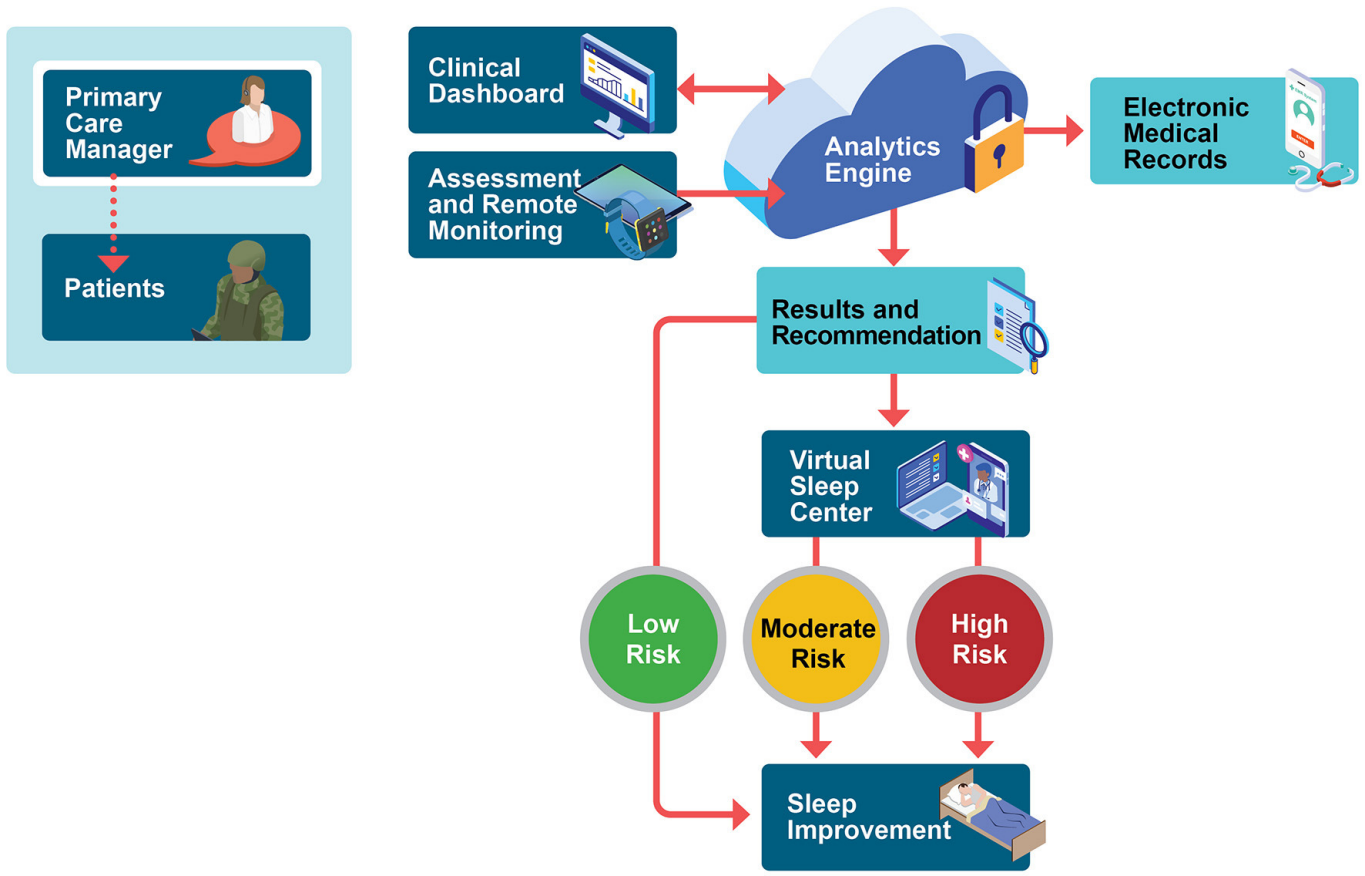
Stakeholder-derived proposed military sleep telehealth workflow. The process begins in the primary care environment, with primary care managers initiating sleep telehealth remote monitoring including commercial-off-the-shelf wrist wearable device and twice-daily symptom surveys. Following the 10-day continuous sleep monitoring period, a secure cloud-based engine provides provisional assessment results and personalized treatment recommendations to patients (in-app) and to providers (via editable reports that can be uploaded into the electronic health record). Population health risk is rated high, medium, or low, and patients are then triaged into an appropriate level of case based on provider-confirmed risk status, provisional diagnoses, and patient preferences. Within-app, patients receive personalized sleep education and training for common sleep problems such as insufficient sleep, insomnia, obstructive sleep apnea, shift work, and nightmares. Remote monitoring and reporting of results are ongoing. Future expansions aim to incorporate a comprehensive virtual sleep center including remote sleep specialist consultation, diagnostic testing, and treatment supported by a human sleep navigator.

**Figure 3:**
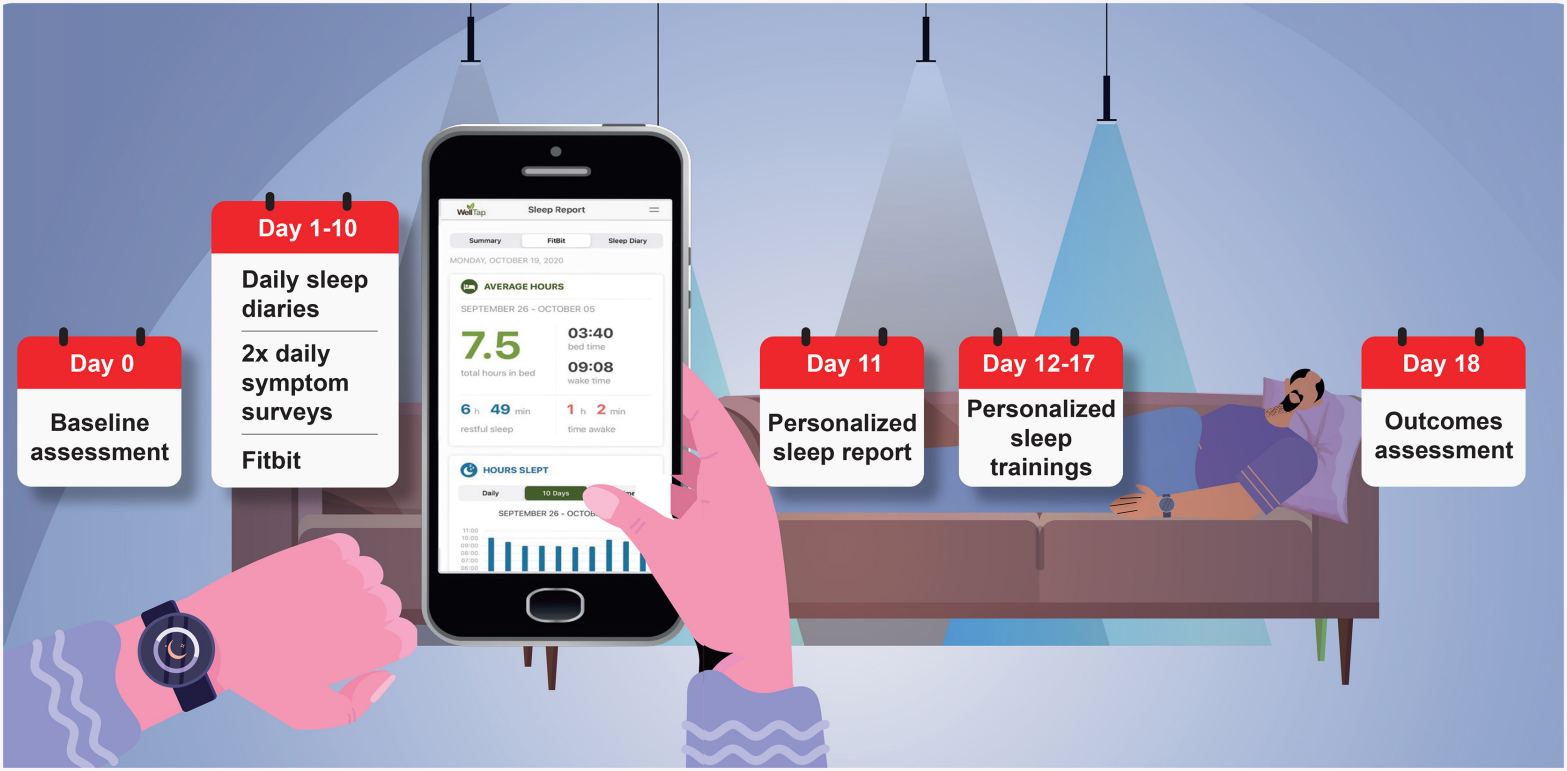
Study timeline for patients with sleep problems. Patients completed a baseline assessment (via WellTap; Day 0); a 10-day intensive remote monitoring assessment and wore a commercial off the shelf sleep tracker (Fitbit Inspire 2; Days 1–10); received a report of assessment results (Day 11); received personalized sleep education and daily sleep training (Days 12–17); and completed a RE-AIM outcomes assessment (Day 18). In parallel, an assessment report was generated for providers (Day 10).

The sleep telehealth platform (WellTap^®^) consists of an online web portal for patients and providers, a secure mobile app (Figure 1), and integrated wearable sensors using a commercial off the shelf sleep tracker. As described elsewhere (Abdelwadoud et al., 2021; Wickwire et al., 2022), the purposes of the sleep telehealth platform are to (1) help PCMs assess sleep complaints, (2) empower patients and PCMs to make evidence-based sleep treatment decisions, (3) deliver evidence-based behavioral sleep treatments via mobile devices, and (4) connect patients with sleep specialists in virtual or physical sleep centers. Figure 2 presents a schematic of the long-term vision for sleep telehealth in the military using this platform. This study focuses on the steps leading to Results and Recommendations as presented in Figure 3.

### Recruitment and study onboarding

This study was originally conceived, proposed, and funded prior to the onset of COVID-19. The original protocol was approved by the WRNMMC IRB and included in-person recruitment (i.e., research staff engaging and supporting referring providers daily and consenting and onboarding participating into the study). However, after the onset of COVID-19 these approaches were no longer feasible. As described elsewhere, the study team worked with funders, the lead and deferring IRBs, and DoD regulators to develop and implement fully remote study procedures (Adornetti et al., 2022). Potential participants were identified by healthcare providers and administrative consult managers at WRNMMC and ATAMMC or self-referred for having sleep problems including insufficient sleep. Each month, the administrative consult managers provided to the study team a list of potential volunteers. Trained research staff contacted these individuals by telephone for eligibility screening. Interested volunteers who were likely to be eligible to participate were then referred to a trained study coordinator to participate in informed consent, receive study instructions, and onboard into the study app (WellTap^®^ downloaded from the Apple store or Google Play store using a secure code). Trained research staff remained available to provide support or answer technical questions throughout the study. All study participants provided informed consent.

### Sleep assessment

At baseline, participants completed a comprehensive assessment including a sleep history intake questionnaire and several validated research questionnaires that are widely used within the DoD to assess sleep and daytime symptoms. Specific research questionnaires assessed symptoms of OSA [Berlin Questionnaire (Netzer et al., 1999)], insomnia [Insomnia Severity Index [ISI] (Morin et al., 1993)], depression [Patient Health Questionnaire-9 (Kroenke et al., 2001)], anxiety [Generalized Anxiety Disorder-7 (Spitzer et al., 2006)], post-traumatic stress disorder (Primary Care PTSD Screen for DSM-5 (Prins et al., 2016) [PC-PTSD]), chronic pain (VA Pain Rating Scale) (Buckenmaier et al., 2013), and history of traumatic brain injury (Brief Traumatic Brain Injury Screen [BTBIS]) (Schwab et al., 2007).

In addition, throughout the 10-day intensive remote monitoring period, participants completed standardized daily sleep diaries each morning and brief symptom surveys administered twice daily (morning and evening). The 10-day duration for the remote monitoring assessment was selected to ensure an adequate sample of sleep and daytime symptoms on both workdays and non-workdays. Sleep diaries assessed time to bed, sleep onset latency, number of awakenings, wake after sleep onset, and subjective sleep quality (scored from 1 to 10, with higher numbers indicating greater perceived sleep quality). Individual symptom survey items assessed mood, cognition, and energy level using an “I feel *tired*” format. For each item, five responses ranged from “not at all” to “very” (scored from 0-4, respectively).

### Commercial sleep tracker

Objective wearable data was collected passively from the Fitbit Inspire 2 device, which uses a triaxial accelerometer to measure ambulatory movement and estimate sleep (Lim et al., 2023). Devices were distributed via priority courier. In order to prevent access to Fitbit sleep data, participants were instructed to remove the sleep tile from the Fitbit application. In addition, participants received written and verbal instructions to turn off alerts in the Fitbit app and to wear the Fitbit device throughout the study (except while bathing or charging the device). Fitbit data was obtained daily in 60 s epochs from Fitbit via application programming interface (API) and integrated directly into the WellTap platform database and analytics engine. This commercial wearable data was subsequently integrated with patient-reported data to deliver evidence-based sleep treatment recommendations and personalized sleep education and trainings.

### Sleep report

Following the 10-day intensive remote monitoring period, patient-reported and objective data were integrated into separate sleep assessment reports for patients and providers. All information was derived from patient self-report or commercial wearable and subsequently processed within the WellTap application. Report content and format were tailored for their respective end users based on insights gained during the earlier qualitative phase of this study (Abdelwadoud et al., 2021; Wickwire et al., 2022). For patients, assessment results were received in-app. The purpose of the patient report was to communicate results from the 10-day remote assessment and to educate and prepare for next steps. For providers, assessment results were available in editable electronic document format (i.e., pdf or docx), so that this could be edited prior to upload in the EHR in the future. The purpose of the provider sleep report was to assist the busy primary care provider in providing evidence-based sleep treatment recommendations, including sleep specialist referral when indicated. As depicted in Table 1, the first page includes summary-level information such as provisional diagnoses (based on patient-reported and wearable-derived data) and recommended next steps based on published clinical practice guidelines. Subsequent pages include more detailed information as might be expected from a specialist consultation. Table 1 presents domains included in patient and provider reports.

**Table 1:** Information content presented in patient and provider assessment reports.

**Patient report (in-app)**	**Provider report**
Sleep assessment results	Summary
Description of possible diagnoses	Population health risks
Key comorbidities	Likely sleep diagnoses
Sleep habits: schedule	Key comorbidities to consider
Sleep habits: pre-sleep routine	Home sleep apnea test eligible?
Sleep habits: environment	Patient preferences and motivation
Sleep habits: sleep thinking	Recommendations
Sleep and wearable device	History of present illness
Sleep diary	Review of systems
Wearable results	Sleep apnea
Recommendations	Circadian rhythm sleep disorder/shift work
Fitbit report	Restless legs syndrome
Total sleep time	Parasomnias
Sleep details	Narcolepsy
Sleep diary	Insomnia
Total sleep time	Psychiatric and daytime sequelae
Sleep details	Past medical history
	Past medical conditions
	Past surgical history
	Current medications
	Medication allergies
	Social history
	Family history
	Questionnaire results
	Self-Reported sleep parameters
	Sleep monitoring
	Sleep diary results
	Wearable results
	Interpretation of discrepancies between sleep diary and wearable device
	Health sleep habits summary
	Sleep schedule and routine
	Bedroom environment
	Sleep beliefs
	Other lifestyle factors

Patients with sleep problems received their personalized sleep assessment results in-app, including integrated results from both patient-reported and COTS wearable data. Provider reports were provided in editable electronic document format.

### Personalized sleep education and training

Based on provisional diagnoses and results of the 10-day remote assessment, patients were assigned personalized sleep education and daily training in multimedia formats, including text, video, and audio, which were delivered and completed within the mobile app. All education and training content followed a modular, sequential approach, such that participants were required to complete the first module in order to access the second module, and so on. To begin, all participants were assigned a Healthy Sleep Habits training suitable for clinical or population health educational efforts. Topics included What Sleep Is, Why Sleep Matters, and How to Get a Good Night Sleep. In addition to this general education, participants were assigned modules based on their personalized assessment results, including disease education and for specific sleep disorders, such as insufficient sleep, OSA, and insomnia. Evidence-based treatment recommendations were based on published clinical practice guidelines. Each morning for 5 days, participants received personalized sleep education messages, which were designed to increase adherence and engagement with the app. For example, messages inquired about completion of educational modules within the app and when needed, provided cognitive-behavioral strategies to increase engagement (e.g., goal-setting, positive self-reward, social support, etc.).

### Implementation outcomes: RE-AIM framework

Implementation outcomes were assessed within the context of the reach, effectiveness, adoption, implementation, and maintenance (RE-AIM) framework (Glasgow et al., 1999; Harden et al., 2018). Reach was defined as the proportion of potential volunteers who were contacted and subsequently enrolled in the study, the proportions of enrolled participants who logged into the app and wore the Fitbit, and the proportion of providers who referred into the study. Effectiveness was defined broadly as satisfaction, with multiple subcomponents (Table 2). Adoption was defined as approval for system-wide adoption. Implementation was defined as adherence. Maintenance was defined as sustainability. Each RE-AIM domain was assessed using objective data (e.g., behavioral adherence) and/or self-report from patients and health care providers. For patients, an established questionnaire (Seligman, 1995) was modified to assess perceptions regarding the personalized sleep report and separately, the personalized sleep education and trainings. For providers a similar, parallel questionnaire was developed to assess perceptions regarding the provider report and separately, the overall sleep telehealth platform. Table 2 summarizes RE-AIM outcome definitions and measures.

**Table 2:** Implementation outcomes and measures mapped on the RE-AIM dimensions.

**RE-AIM dimension**	**Definition**	**Measure**
Reach	*Utilization* Proportion of potential volunteers who were contacted and elected to enroll in the study Proportion of participants who logged into the app and who wore the Fitbit Proportion of providers who referred into the study	Data tracking
Effectiveness	*Satisfaction* Usability Perceived improvement Perceived risks and benefits Perceived credibility Overall satisfaction	Patient questionnaire; Provider questionnaire
Adoption	*Approval for system-wide use* Proportion of patients who rate the platform as acceptable for system-wide adoption Proportion of providers who rate the platform as acceptable for system-wide adoption	Patient questionnaire; Provider questionnaire
Implementation	*Adherence* Proportion of participants who (1) complete the baseline assessment, (2) complete daily sleep diaries, (3) complete twice daily symptom surveys, (4) review personalized sleep report, (5) engage with app for personalized sleep education and training	Data tracking
Maintenance	*Sustainability* Proportions of participants who choose to recommend the app to others and to continue to use the app themselves Proportion of providers who choose to include the platform in their practice	Patient questionnaire; Provider questionnaire

For effectiveness, patients were queried regarding the personalized sleep report and separately, personalized sleep education and training. Providers were queried regarding the provider report and assessment, treatment planning, and documentation.

## Results

### Demographic and military characteristics

Participants (*N* = 270, 55.2% men, mean age = 45.8 [SD = 13.0] years) included active duty (46.8%), retired military (27.4%), or civilian (24.8%) adults with sleep complaints who were recruited remotely and/or in-person. Participants self-identified as White (56.3%), Black (23.7%), Hispanic (8.5%), or Asian (7.0%) race/ethnicity. Table 3 presents demographic and military characteristics of the sample.

**Table 3:** Summary of participant demographic and military characteristics (*N* = 270).

		***n***	**%**
Sex	Male	149	55%
	Female	121	45%
Race/ethnicity	White	152	56%
	Black	64	24%
	Hispanic/Latino	23	9%
	Asian	19	7%
	Native American/Alaskan Native	7	3%
	Other	5	2%
Military status	Active-duty	111	41%
	Retired	74	27%
	Activated Reserve	11	4%
	Activated National Guard	7	3%
	Civilian	67	25%
Military rank (Enlisted paygrade)	E3	6	5%
	E4	16	13%
	E5	20	16%
	E6	8	7%
	E7	14	11%
	E8	3	2%
	E9	2	2%
	W3	3	2%
	W4	1	1%
	O2	7	6%
	O3	6	5%
	O4	11	9%
	O5	22	18%
	O6	4	3%

### Sleep and daytime symptoms

For research questionnaires, all results are reported using established cut points. Based on responses to the Berlin Questionnaire, 65.6% of participants were found to be at high-risk for OSA. Based on the ISI, 38.2% of participants reported moderate to severe insomnia. Based on the ESS, 38.5% of participants reported moderate to severe excessive daytime sleepiness. Based on the GAD-7, 14.1% of participants reported moderate to severe anxiety symptoms. Based on the PHQ-9, 20.4% of participants reported moderate to severe depressive symptoms. Based on the PC-PTSD, 20.4% of the sample reported likely PTSD. Based on the BTBIS, 10.4% of participants reported a likely deployment-related TBI. Table 4 summarizes sleep and daytime symptoms.

**Table 4:** Summary of symptom severity based on validated questionnaires (*N* = 270).

**Measure**	**Cutoff**	***n***	**%**
Insomnia Severity Index	No insomnia (0–7)	53	19.5%
	Subthreshold insomnia (8–14)	114	42.2%
	Moderate insomnia (15–21)	87	32.2%
	Severe insomnia (22–28)	16	5.9%
Epworth Sleepiness Scale	Normal (0–9)	166	61.5%
	Excessive daytime sleepiness (10–15)	71	26.3%
	Severe daytime sleepiness (16–24)	33	12.2%
Patient Health Questionnaire-9	Minimal depression (0–4)	113	41.9%
	Mild depression (5–9)	102	37.8%
	Moderate depression (10–14)	39	14.4%
	Moderately severe depression (15–19)	14	5.2%
	Severe depression (>20)	2	74.0%
Generalized Anxiety Disorder-7	No anxiety (0–4)	164	60.7%
	Mild anxiety (5–9)	68	25.2%
	Moderate anxiety (10–14)	24	8.9%
	Severe anxiety (>15)	14	5.2%
Primary Care PTSD Screen DSM-V	Probable PTSD (cutoff >3)	56	20.7%
Brief Traumatic Brain Injury Screen	Likely mTBI	28	10.4%

### Sleep parameters

Table 5 summarizes results from sleep diary and commercial wearable sleep tracker during the 10-day intensive remote monitoring assessment. Based on sleep diaries, participants slept an average of 6.6 (SD = 1.8) h, with a sleep onset latency of 30.1 (SD = 39.3) min and wake after sleep onset of 22.9 (SD = 35.2) min. Mean sleep efficiency was 84% (SD = 13), and subjective sleep quality was 6.1 (SD = 2) on a scale from 1 to 10. Based on Fitbit, total sleep time was 6.1 (SD=1.8) h. Total wake time was 51.0 (SD = 22.0) min, and mean sleep efficiency was 88.0% (SD = 4). The correlation between 10-day sleep diary and Fitbit TST was *r* = 0.43 and between 10-day sleep diary and Fitbit SE was *r* = 0.15.

**Table 5:** Summary of sleep continuity as assessed via sleep diary (*n* = 270) and Fitbit (*n* = 251) during 10-day remote monitoring assessment.

**Sleep parameter**	***M***	**SD**	**Range**
**Sleep diary**			
Total sleep time (hours)	6.6	1.8	0, 13.0
Sleep onset latency (minutes)	30.1	39.3	0, 480
Wake after sleep onset (minutes)	22.9	35.2	0, 330
Sleep efficiency	0.84	0.1	0.01, 1
Sleep quality	6.1	2.0	1, 10
**Fitbit**			
Total sleep time (hours)	6.1	1.8	0.41, 14.1
Total wake time (minutes)	51.0	22.0	0, 264
Sleep efficiency	0.88	0.04	0.31, 1

### Implementation outcomes

#### Reach

The research team attempted to contact 839 potential volunteers, of whom 426 (50.8%) were contacted successfully. Of contacted volunteers, 294 (69.0%) chose to enroll in the study. Of enrolled participants, 288 (98.0%) logged into the app and 278 (94.6%) wore the Fitbit.

#### Effectiveness

Table 6 summarizes effectiveness results for patients (*N* = 251). Nearly nine in ten participants found the sleep report and sleep education very usable, mostly usable, or usable (85.2% for sleep report and 87.2% for sleep education, respectively). Similarly, 82.8% of participants rated the sleep report as very credible, mostly credible, or credible, and a comparable percentage (86.4%) rated the sleep education and trainings similarly.

**Table 6:** Summary of participant perceived effectiveness results (*N* = 251).

		**Not at all**	**A little**	**[Term]**	**Mostly**	**Very**
**Sleep report**						
	How **usable** is the sleep report?	5.2%	8.5%	34.0%	26.7%	18.5%
	How much does the sleep report **improve** your understanding of your sleep problems?	7.8%	17.4%	29.6%	20.0%	18.2%
	How **credible** to you are the results presented in the sleep report?	4.1%	11.9%	37.8%	20.4%	18.9%
	How **acceptable** would it be for the hospital (or DOD) to adopt this sleep report throughout the system?	4.8%	8.9%	37.4%	18.9%	23.0%
	Overall, how **satisfied** are you with the sleep report?	4.8%	16.3%	35.6%	21.9%	14.4%
**Sleep education**						
	How **usable** are the in-app sleep education and trainings?	4.8%	7.0%	35.2%	21.5%	24.4%
	How much do the in-app sleep education and trainings **improve** your sleep problems?	14.4%	38.9%	20.0%	9.6%	10.0%
	How **credible** to you are the in-app sleep education and trainings?	4.4%	8.2%	34.4%	20.4%	25.6%
	How **acceptable** would it be for the hospital (or DOD) to adopt the in-app sleep education and trainings?	4.4%	7.4%	33.0%	17.0%	31.1%
	Overall, how **satisfied** are you with the sleep report?	4.8%	16.3%	35.6%	21.9%	14.4%

“Term” indicates the anchor term for a given item, e.g., usable, credible, acceptable, etc.

Table 7 summarizes effectiveness results for providers (*N* = 43). Greater than nine in ten providers rated the sleep report very usable, mostly usable, or usable for assessing sleep problems (93%), providing evidence-based sleep treatment recommendations (90.8%), and documenting sleep problems (93%). Similarly, nine in ten providers perceived that the sleep report would very much improve, mostly improve, or improve assessment of sleep problems (90.8%), sleep treatment planning (88.4%), and documentation (90.8%).

**Table 7:** Summary of provider effectiveness results (*N* = 43).

		**% Not at all**	**% A little**	**% [Term]**	**% Mostly**	**% Very**
Usability	How **usable** is the sleep report for assessing sleep problems among patients you see?	2.3	2.3	37.2	30.2	25.6
	How **usable** is the sleep report for providing evidence-based treatment recommendations (treatment planning)?	2.3	2.3	32.6	32.6	25.6
	How **usable** is the sleep report for documenting sleep problems?	0	4.7	34.9	39.5	18.6
Acceptability	How **acceptable** would it be for the hospital (or DoD) to adopt this sleep telemedicine platform throughout the system?	2.3	9.3	39.5	23.3	23.3
Perceived improvement	How much would the sleep report **improve** your assessment of sleep problems?	2.3	7.0	25.6	32.6	32.6
	How much would the sleep report **improve** your evidence-based sleep treatment recommendations (treatment planning)?	2.3	9.3	37.2	23.3	27.9
	How much would the sleep report **improve** your documentation of sleep problems?	2.3	7.0	25.6	32.6	32.6
Credibility	How **credible** to you are the sleep assessment results in the sleep report?	0	11.6	48.8	20.99	11.6
	How **credible** to you are the evidence-based sleep treatment recommendations in the sleep report?	0	11.6	37.2	25.6	18.6
Overall satisfaction	Overall, how **satisfied** are you with the content of the report?	0	9.3	25.6	39.5	25.6
	Overall, how **satisfied** are you with the format of the report?	0	9.3	25.6	32.6	30.2
	Overall, how **satisfied** are you with the length of the report?	0	14.0	37.2	25.6	25.6

“Term” indicates the anchor term for a given item, e.g., usable, credible, acceptable, etc.

#### Adoption

Nearly nine in ten patients reported that it would be very much acceptable, mostly acceptable, or acceptable for the DoD to adopt the sleep telehealth platform throughout the health system, including the sleep report (85.2%) and sleep education (87.2%). Similarly, 86.1% of providers reported that it would be very much acceptable, mostly acceptable, or acceptable for the hospital (or DoD) to adopt the sleep telemedicine platform throughout the system. Results are presented in Table 6 (patients) and Table 7 (providers).

#### Implementation

One hundred percent of participants completed the baseline assessment and reported wearing the Fitbit device. However, for 5.2% of participants, Fitbit data was unavailable due to changes within Fitbit permissions for these users. During the 10-day intensive remote monitoring period, the mean number of sleep diaries completed was 9.3 (SD = 1.3) of 10 possible, and the mean number of mood/daytime function surveys was 18.6 (SD = 2.5) of 20 possible. Two hundred fifty-one (93.0%) of participants completed the RE-AIM outcomes assessment. Results are presented in Table 6.

#### Maintenance

Eighty-two percent (82.0%) of participants reported that they were likely, mostly likely, or very likely to recommend the sleep app to others, and a higher number (92.4%) wanted to continue to use the app themselves. Most providers (86%) reported that they were very likely, mostly likely, or likely to continue to use the platform in their practices.

## Study 2

### Overview

#### Focus groups with patients with sleep problems

Following the clinical implementation study, participants with sleep problems were invited to participate in focus group. Four focus groups (*N* = 21 patients) were led by an experienced military qualitative researcher (RJ) and supported by trained research staff. Focus groups were conducted remotely via secure video conferencing platform, with multiple measures to protect anonymity, and lasted < 60 min. The focus group guide developed for this study covered (1) experiences participating in the study, (2) review of preliminary study results, and (3) suggestions for future research directions. All sessions were audio recorded, transcribed, and de-identified, and transcripts were reviewed to identify common themes.

#### Key informant interviews with health care providers and economic stakeholders

PCMs (*n* = 8) and economic stakeholders (*n* = 4) were recruited for one-on-one, semi-structured key informant interviews via word of mouth. Interviews were conducted remotely by an experienced qualitative researcher (DCW) and lasted < 30 min. Using an interview guide developed for this study, topics included (1) review of preliminary study results, (2) suggestions for future research, and (3) factors that would influence future purchasing decisions. Recording, transcription, and data analysis procedure mirrored those described above.

### Qualitative results

#### Focus groups with patients with sleep problems

Table 8 presents results of the qualitative analysis of focus group data. Participants perceived benefits including finding the mobile app easy to use and helpful in monitoring their sleep, developing more awareness of their sleep behaviors and curiosity about improving sleep, and finding the sleep report beneficial to share with their medical providers. Challenges to participation included perceived issues with the Fitbit, finding some survey questions on the app challenging to answer categorically, and a desire for stronger subjective sleep improvement outcomes. In terms of study improvement and future research, participants strongly supported a virtual sleep center using a sleep navigator as well as recommendations for additional sleep treatment resources in the app-based learning library. Improving sleep awareness and focusing on time efficiency were suggested as recruitment tools for future studies.

**Table 8:** Key themes and illustrative quotations from focus groups regarding participation in study.

**Theme**	**Illustrative quotation**
**Benefits of participation**	
The mobile application was easy to use and helpful.	And I thought it [participation] was really helpful. I thought it was easy to do using the app, so I thought it was good when I was going through it.
Participation led to reflection, better awareness, and curiosity about improving sleep	But what it did for me was enhance my curiosity about reading more about a study about what possible solutions can be found for sleeplessness, and so I got into yoga with mindfulness. And I read reports about nutrition, what foods may affect sleeping which way.
The sleep report was viewed as a helpful resource to share with medical providers to improve care	I like the provider pieces of it that helps them do their job because … for active duty it's hard to get the same doctors and for retired people like myself—and I know others it's even harder to get the same doctor and the same provider—so I think having a copy to be able to share with whichever provider is very useful.
**Challenges of participation**	
Some participants reported concerns about the Fitbit, such as questioning its accuracy, wearing discomfort, or forgetting that it's not waterproof.	And the only issue I had is the device was not accurate with other devices I use. I actually checked them and it was on average—the minimum was 10% off or heart rate. So that was something I did notice…
Categorical survey questions were sometimes viewed as repetitive and difficult to answer	I would just offer a suggestion… maybe you could just have some questions on a continuum… that was 1 through 10. You know you're a 2.5, you're a 6.5, you're whatever along that scale. It might be easier than trying to jam it into a specific category.
Some participants' sleep did not improve as much as desired.	And in the past few months, my sleep has improved but not significantly.
**Suggestions for improving the study and future research**	
Future research steps, such as including a Sleep Navigator, were seen as beneficial	Yeah, it [having a Sleep Navigator] would have put my mind at ease instead of me trying to Google search what does this mean, what does that mean? So if someone could actually explain everything that that would be awesome.
Participants recommended including more sleep and mental health resources in the mobile application.	I was thinking at the time it would be helpful if there was even just a reference to where we could find more information about how to be successful with CPAP. Because my appointments for my follow-up were 6 months or 4 months out and then they're just like, “Hey, how's it going with your CPAP?”
For marketing strategies, participants recommended focusing on time efficiency and improved awareness of sleep-related behaviors.	It gives you not just the patient, but also the provider, the tools to put in their toolbox to manage their sleep study, to understand what they're going through, to give them suggestions on how to correct it. It almost gives gave me the opportunity to take charge.

#### Key informant interviews with primary care managers and economic stakeholders

Table 9 presents results from the one-on-one interviews with PCMs and economic stakeholders. Perceptions regarding preliminary results focused on high study credibility due to strong adherence rates, viewing positive outcomes as the most important variable, the necessity of finding the report to be quickly understandable and implementable, and the importance of increasing access to sleep care in the MHS. Suggestions for further research include the importance of integrating results into the electronic medical record for easy accessibility. A virtual sleep center and sleep navigator support were viewed as invaluable additions to current military resources. Factors influencing future purchasing focused on demonstrating cost savings, objective outcomes, and efficient utilization of resources.

**Table 9:** Summary of key themes and illustrative quotations from primary care managers (PCMs) and economic stakeholders regarding study results and future directions.

**Themes**	**Illustrative quotation**
**Perceptions of preliminary research results**	
Participants' high adherence rates were indicative of a credible and helpful treatment.	The good adherence [rates from participants] makes me think that these patients were really excited about this option and doing it this way.
PCMs viewed positive Improvement rates as the most important outcomes	It should be usable and acceptable, but the bottom line is, does it actually improve sleep?
PCMs' viewed the ability to quickly understand and implement recommendations from the reports as the most important factor.	Primary care has so many different requirements on their time. If it's not user friendly, if it if it's an extra step it is not going to be used and so won't be useful.
Providing better access to sleep treatment and maximizing resources were seen as highly valuable.	Maximizes access to care with limited resources [using this as a] screening tool for if somebody's coming in with kind of broad or vague sleep complaints to allow them to have some form of assessment without necessarily taking up the resource that is our Sleep Medicine providers to evaluate them—that is one really big utility in this.
**Suggestions for further research and implementation**	
Initiating and finding results from referral to this program were the most important factors for future implementation	When I put in a referral, what do I need to do to get that referral and how much work is it for me and my team to make that happen? And then on the results side too, how do we close that loop? Because I placed a referral now… you never know if someone did their referral… I just want to be able to get that information.
The virtual sleep center and sleep navigators were seen as valuable additions to stakeholders' current practices	And I think especially when it comes to sleep treatment for insomnia it is just so difficult. So, if you have some kind of accountability system supporting your treatment plan, I think it's very helpful.
**Factors that would influence purchasing decisions**	
Demonstrating objective outcomes, cost savings, and improved care utilization were viewed as important future implementation factors	If I was going to take this to my hospital leadership… I think being able to show them they did a study where… a large number of people enrolled…they had incredible results… I think that the improvement number probably needs to be higher if you want the military to spend money on it.
Military-related challenges to purchasing wearable devices were seen as a major obstacle to implementation	I think the big burden you'll have… is purchasing with TRICARE trying to purchase a Fitbit or any type of equipment for a single person is incredibly difficult and harder than it should be.

## Discussion

In this study, a novel sleep telehealth platform including a web-based portal, native mobile apps, and integrated commercial wearable sensors was successfully implemented at two MTFs. Our mixed-methods approach was driven by continuous engagement with key stakeholders with at-times competing interests, including patients with sleep problems, PCMs, and economic decision-makers. Quantitative measures of behavioral adherence among patients, RE-AIM implementation outcomes among both patients and providers, and qualitative feedback from all stakeholder groups, support ongoing advancement of sleep telehealth to increase access to high-quality sleep medicine care throughout the U.S. military.

By any standard, insufficient and disturbed sleep as well as clinical sleep disorders such as insomnia and OSA represent major threats to force readiness and long-term health among MHS beneficiaries. Yet the MHS faces a dramatic challenge in limited access to care, resulting in excessive leakage to local TRICARE networks (i.e., standard purchased care). Technology and telehealth represent vital and underutilized levers to increase access to high-quality, cost-effective care. In sleep medicine, as in other areas of medicine, a technology and telehealth boom is being driven by technological advancements that support telehealth and remote monitoring, patient preferences for relative convenience and time efficiency, and health system emphasis on economic value. It is thus not surprising that telehealth approaches are increasingly deployed to improve screening, assessment, diagnosis, and treatment, including remote delivery of care. The current project represents an important, foundational effort to harness these forces to increase access to high-quality sleep medicine care in the MHS.

As evidenced by our results pertaining to reach, interest in our sleep telehealth study was very high. Of potential volunteers who were contacted successfully, nearly 70% elected to participate in the study. In addition, effectiveness was rated very highly by both patients (for understanding sleep problems) and providers (for improving care delivery). Likewise, adoption was notably high, with the vast majority of patients and PCMs reporting strong support for system-wide adoption of the sleep telehealth platform. Implementation, defined as behavioral adherence, was also high, with the vast majority of participants completing all aspects of the study. Finally, maintenance was rated highly by both patients and PCMs, who would continue to use the app and overall platform, respectively.

At the same time, two exceptions to these uniformly positive results warrant consideration. First, although participants were highly satisfied with the remote monitoring assessment, sleep education and trainings were rated less highly. We attribute this discrepancy to our study design and instructions that we provided to participants. Because the primary objective of this study was to evaluate the remote monitoring assessment of sleep complaints, participants were provided detailed instructions regarding how to complete the sleep diaries and daily symptom surveys. On the other hand, our objective for the personalized sleep education and trainings was naturalistic observation. That is, we wanted to observe how participants would interact with the app during a subsequent brief “open use” period. Participants were not provided any specific instruction regarding how to complete personalized sleep education and trainings. In retrospect it is thus not surprising that participants reported less clarity regarding this aspect of the study, including lower perceived improvement reported in the RE-AIM survey as well as qualitative focus groups. In this vein, an important lesson from this study is to delineate very clearly assessment vs. treatment components and to educate patients regarding key next steps and expectations. In addition to sleep education and trainings, qualitative feedback regarding the Fitbit was mixed, suggesting that future research should further examine potential benefits of including commercial wearable data in sleep medicine clinical care.

Throughout all three phases of this mixed-methods study, patients, PCMs, and economic stakeholders identified challenges with current approaches to sleep management in the MHS and reported enthusiasm for sleep telehealth as an evidence-based alternative that could increase access to care, improve patient and provider experience, and improve military-relevant outcomes including readiness and long-term health. In terms of optimizing implementation, including a human “sleep navigator” emerged as a consistent request and recommendation. Patients and PCMs requested that this sleep navigator guide and support patients throughout the sleep treatment process and serve in a physician-extender capacity to help manage many routine clinical tasks including patient education and support. Several studies have examined the potential for non-sleep specialists to increase access to care and deliver high-quality care (Kunisaki et al., 2018; Van Ryswyk et al., 2022). For example, non-physician providers have been included in delivery of high-quality OSA care (Antic et al., 2009; Pendharkar et al., 2019), and cloud-based sleep coaches have effectively increased PAP adherence among veterans (Alessi et al., 2016). In terms of web-based and mobile health CBTI in particular, an ample literature supports the role of non-sleep specialists (Kaldo et al., 2015; Kemper and Khirallah, 2015; Lancee et al., 2016; Beukes et al., 2018; Krieger et al., 2019). Clearly, the role for a sleep navigator warrants further investigation in support of the DHA Quadruple Aim: improved health readiness, better health, better care, and lower cost.

Our study possesses strengths. First, our research question was timely and highly relevant within the MHS and sleep medicine more broadly. Recent decades have seen an explosion of interest in sleep and sleep disorders, yet in the MHS and in the civilian sector, demand for care greatly exceeds available supply. Second, a related strength is that we purposefully recruited individuals with a very broad range of sleep complaints, including insufficient sleep. Whereas the vast majority of prior studies have focused on patients experiencing a narrow range of sleep problems, our study aimed to screen, assess, educate, and triage patients with a very broad range of sleep complaints—what we believe is the optimal approach to drive population health and ensure efficient allocation of resources. Third, our approach to implementation was driven by continuous engagement with diverse stakeholders with at-times competing interests. In our experience within the MHS and other health systems, most implementation efforts fail not due to low merit but due to lack of alignment and shared vision among these diverse stakeholder groups. Present results provide a meaningful road map to guide engagement with stakeholders to ensure successful implementation in the future. Third, our assessment of implementation results was comprehensive, including measures of both behavioral adherence as well as patient-reported and PCM-reported outcomes within the established RE-AIM framework. Finally, our team successfully responded to challenges related to COVID-19 that could not possibly have been foreseen, resulting in an improved project that advanced our efforts one step closer to creation of a “virtual sleep center” to deliver evidence-based sleep medicine care MHS beneficiaries throughout the US and across the globe.

At the same time, results of our study should be interpreted in light of several important limitations. First, our study focused on remote assessment and personalized sleep education and trainings only. In terms of remote assessment, incorporation of commercial wearable data adds an important objective measure of sleep (Kang et al., 2017; Kubala et al., 2020; Stone et al., 2020; Chinoy et al., 2023). However, commercial wearable data has not yet been validated for clinical use, and a number of participants reported dissatisfaction with having to wear a commercial sleep tracker device as part of this study. In terms of personalized sleep education and trainings, we observed engagement during a five-day period but did not attempt to treat specific sleep disorders. Such efforts will be vital in future studies. Second, participants were recruited from only two MTFs in one geographic region with ample local sleep resources. Although the military and civilian composition of our sample was typical for patients at WRNMMC and ATAMMC, it is unknown how well results will generalize to MTFs in other regions or areas with fewer local sleep resources. This being said, our fully remote approach and proposed future “virtual sleep center” do not require local sleep resources. Indeed, a fully virtual approach could be used to increase access to high-quality sleep medicine care throughout the U.S. and worldwide, while benefitting from economy of scale to support cost-effective use of resources.

Our results suggest several important directions for future research. In this study participants were assigned personalized sleep education and training based on results of the 10-day intensive remote monitoring assessment, including patient preferences for care. This approach holds great potential to triage patients to appropriate levels of evidence-based care, including sleep specialist consultation when indicated. Thus, our most important recommendation is that future studies should seek to examine health and systems outcomes associated with each personalized sleep care pathway, including impact on routine clinical care. Second, further evaluation of wearables is indicated. Despite the obvious appeal and potential advantages of passive data collection, feedback regarding the wearable was mixed, with some patients as well as some PCMs questioning validity or applicability of wearable data. Given that optimal approaches to leveraging commercial wearable-derived sleep data for clinical care have not yet been determined, further investigation of how to integrate wearables is warranted. Third, our novel remote monitoring assessment provides a wealth of information captured dynamically over 10 days. This information can be used to identify individuals at risk for adverse outcomes, to predict response to sleep treatment, or to enrichen the assessment of sleep treatment outcomes. Future studies should seek to incorporate this information into analytic frameworks to increase personalized care delivery and improve outcomes. Fourth, future implementation of sleep telehealth should build upon present findings, including integration with the EHR and especially incorporation of a human sleep navigator to support and guide patients throughout the sleep treatment process. Patients and PCMs lamented the degree to which sleep patients must navigate a complex sleep care system, including PCMs, sleep specialists, and durable medical equipment (DME) providers. A human sleep navigator was identified as a vital approach to guide and support patients through this process. Fifth, future research should also consider differences and similarities between military and civilian health systems. For example, from a clinical perspective, military personnel often experience sleep disorders at younger ages, with less obesity, and without major medical comorbidities common in civilian populations. From a systems perspective, not only is the MHS a major global enterprise but is also currently undergoing a major technology led intervention, including revamping the EHR (>$10 billion). Increased military-civilian collaboration is one way to share “lessons learned” to optimize outcomes for all stakeholders, most important patients. Finally, in the modern economic climate of increasing costs on the one hand and limited resources on the other, future research should quantify potential economic benefit from sleep telehealth.

In conclusion, patients with sleep problems, PCMs, and economic stakeholders were highly enthusiastic about this foundational effort and also offered helpful suggestions to optimize future implementation. Clearly, sleep telehealth and mobile health represent promising approaches to increase access to cost-effective, evidence-based care for sleep disorders in the U.S. military. Results of this study strongly support continued examination of sleep telehealth and related issues in the MHS.

## Data availability statement

The datasets presented in this article are not readily available because consistent with the policies of Human Research Protections Program in the Department of Research Programs at Walter Reed National Military Medical Center, any request for raw data will require a data sharing agreement (and protocol modification, if applicable) to limit the use of data and to protect participant confidentiality. Any recipient of a limited, deidentified dataset will be prohibited from identifying or reidentifying any participant whose data are provided. Requests to access the datasets should be directed to EW, ewickwire@som.umaryland.edu.

## Ethics statement

The studies involving humans were approved by Walter Reed National Military Medical Center. The studies were conducted in accordance with the local legislation and institutional requirements. The participants provided their written informed consent to participate in this study.

## Author contributions

EW: Conceptualization, Funding acquisition, Investigation, Methodology, Project administration, Supervision, Writing—original draft, Writing—review & editing. JC: Conceptualization, Funding acquisition, Investigation, Methodology, Project administration, Supervision, Writing—original draft, Writing—review & editing, Data curation, Formal analysis. VC: Conceptualization, Investigation, Methodology, Project administration, Supervision, Writing—review & editing, Funding acquisition, Writing—original draft. SA: Data curation, Investigation, Methodology, Project administration, Supervision, Writing—review & editing. RJ: Data curation, Investigation, Methodology, Project administration, Supervision, Writing—review & editing. SW: Conceptualization, Investigation, Methodology, Writing—review & editing, Project administration, Supervision. CT: Methodology, Project administration, Supervision, Writing—review & editing. DW: Data curation, Formal analysis, Investigation, Methodology, Writing—review & editing. JA: Conceptualization, Data curation, Formal analysis, Funding acquisition, Investigation, Methodology, Writing—original draft, Writing—review & editing.

## Footnotes

^1^News HI. Available online at: https://www.healthcareitnews.com/news/himss23-dha-developing-virtual-first-health-ecosystem
